# Boosting the electrochemiluminescence of luminol by high-intensity focused ultrasound pretreatment combined with 1T/2H MoS_2_ catalysis to construct a sensitive sensing platform

**DOI:** 10.1016/j.ultsonch.2022.106264

**Published:** 2022-12-12

**Authors:** Lin Du, Huixin Zhang, Zhenyu Wang, Tingting Zhuang, Zonghua Wang

**Affiliations:** College of Chemistry and Chemical Engineering, Shandong Sino-Japanese Center for Collaborative Research of Carbon Nanomaterials, Instrumental Analysis Center of Qingdao University, Institute of Biomedical Engineering, Qingdao University, Qingdao, Shandong 266071, China

**Keywords:** Electrochemiluminescence, High-intensity focused ultrasound, Pretreatment, 1T/2H MoS_2_, Luminol-O_2_

## Abstract

In the luminol-O_2_ ECL system, O_2_ as an endogenous coreactant has the advantages of non-toxicity and stability. Improving the efficiency to generate radicals of O_2_ is a challenge currently. In this work, a strategy combining physical method - ultrasound and nanomaterial with unique physicochemical properties was designed to enhance the ECL signal of luminol-O_2_ system. Specifically, high-intensity focused ultrasound (HIFU) pretreatment as a non-invasive method could generate ROS (H_2_O_2_, O_2_^•−^, OH•, ^1^O_2_) in situ, triggering and boosting the ECL signal of luminol. In addition, 1T/2H MoS_2_ with excellent catalytic activity could catalyze the H_2_O_2_ produced in situ, accelerate the oxidation of luminol and further enhance the ECL response. At the same time, combined with the catalytic hairpin assembly (CHA) reaction, the constructed ECL biosensing platform showed excellent performance for the detection of miRNA-155. The concentration range of 0.1 fM ∼ 1 nM with the detection limit as low as 0.057 fM were obtained. Furthermore, the ECL biosensor was also successfully applied to the determination of miRNA-155 in human serum samples. The established ECL sensing platform opens up a promising method for the detection of clinical biomarkers.

## Highlights

• A novel strategy combining ultrasound with nanomaterial was designed.
• High-intensity focused ultrasound (HIFU) could generate H_2_O_2_ and O_2_^•−^ in situ.
• 1T/2H MoS_2_ could catalyze the H_2_O_2_ to boost the ECL signal of luminol-O_2_ system.
• The ECL biosensor was successfully applied to the determination of miRNA-155.

## Graphical abstract

High-intensity focused ultrasound pretreatment as a non-invasive method could generate H_2_O_2_ and O_2_^•−^ in situ, triggering and boosting the ECL signal of luminol-O_2_ system. Meanwhile, 1T/2H MoS_2_ could catalyze the H_2_O_2_ formed in situ to enhance the ECL response.

**Figure:**
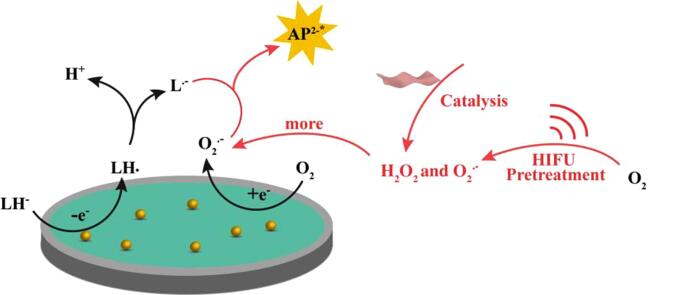

## 1 Introduction

Electrochemiluminescence (ECL) combines both electrochemical methods and chemiluminescence, which has the advantages of low background signal, wide dynamic range, high sensitivity, simple instrument and low cost [1], [2], [3], [4], [5]. Luminol has been extensively used in the field of ECL sensing because of its high luminous rate, non-toxic and low cost [6], [7], [8]. O_2_ as an endogenous coreactant has the characteristics of mild reaction and convenient operation, and the derived radicals could increase the ECL efficiency of luminol based system [9]. However, the low decomposition rate of O_2_ usually could not produce adequate intermediate reactive oxygen species (ROS) to obtain the ideal signal in luminol-O_2_ ECL system [10]. Researchers have introduced a third substance, coreaction promoter, to improve the free radical yield of O_2_. For example, (i) Fe@Fe_2_O_3_ nanowires [11], CeO_2_/SnS_2_ heterostructure [12], ZnO nanostars [13], Cu-doped TiO_2_ [14] and other nanomaterials were introduced into lumino-O_2_ system as the coreaction promoter to amplify the ECL signal; (ii) high-entropy oxides containing five metal components (Ni, Co, Cr, Cu and Fe) have made important contributions in catalyzing the conversion of dissolved O_2_ and improving the ECL efficiency of luminol [15]. Despite the extensive work that has been done so far, it remains challenging to develop a simple and efficient method to improve ECL signal in the luminol-O_2_ system.

High-intensity focused ultrasound (HIFU), as a non-invasive means, has attracted more and more attention in clinical application [16], [17], [18]. The cavitation bubble is generated by the high negative pressure in the focal region of HIFU, and then oscillated and collapsed by HIFU. When the bubble shrinks, water molecules or O_2_ are thermically decomposed due to high temperature and pressure, and then the ROS are generated containing H_2_O_2_, superoxide anion radical (O_2_^•−^), hydroxyl radical (OH•), singlet oxygen (^1^O_2_) [19]. ROS produced by HIFU is highly oxidized, which can induce apoptosis of cancer cells and treat cancer safely and effectively [20], [21]. So far, there have been only a few studies on HIFU/ECL published, while continuous low-frequency ultrasound has been applied to ECL. For example, it has been proved that ultrasound irradiation could markedly enhance the ECL emission of arylacetate [22]. In addition, ECL response of Ru(bpy)_3_^2+^/lidocaine system was also obviously improved with ultrasound irradiation [23]. Inspired by the above, HIFU pretreatment, as a non-invasive pretreatment means, was introduced into the lumino-O_2_ ECL system to promote the conversion of dissolved O_2_ to ROS in situ and enhance ECL signal firstly in our group [24].

MoS_2_, as one of the most typical transition-metal disulfides (TMDCs) with two-dimensional ultrathin atomic layer structure, has been widely investigated in supercapacitor and catalysis due to its excellent layered structure and electronic property [25], [26], [27]. For example, the narrow band gap of MoS_2_ endows it have photoelectric effect [28], thus, it is promising in photocatalysis [29], [30]. Furthermore, MoS_2_ shows electrocatalytic activity because of unique electronic structure, and it has been expected to be a wonderful catalyst for hydrogen evolution reaction (HER) [31], CO_2_ [32] or N_2_ [33] reduction reactions. MoS_2_ has various crystal phase structures due to different coordination of atoms [34]. 2H MoS_2_ crystal structure have poor conductivity, while 1T MoS_2_ is a metallic phase with a higher catalytic performance than the 2H MoS_2_ owing to that it has active sites at the base plane and edge. Nonetheless, 1T MoS_2_ has some problems such as strict synthesis conditions and poor stability. Therefore, the catalytic activity of 1T/2H MoS_2_ is higher than 2H MoS_2_, and its thermochemical stability is better than that of 1T MoS_2_, which has great research potential.

In the luminol-H_2_O_2_ system, the electrocatalytic activity of MoS_2_ nanosheets was developed for the decomposition of H_2_O_2_, which could accelerate the oxidation of luminol and enhance the ECL emission. Jia’s group synthesized MoS_2_-PEI-Au nanocomposites which significantly amplified the ECL sensing signal [35]. Wei’s group used the good catalytic effect of MoS_2_ nanoflowers to accelerate the decomposition of H_2_O_2_, increase the ECL intensity of luminol, and improve the sensitivity of quenching sandwich-type immunosensor [36]. Notably, 1T/2H MoS_2_ that has higher electrocatalytic activity is rarely applied in ECL field.

**Scheme 1:**
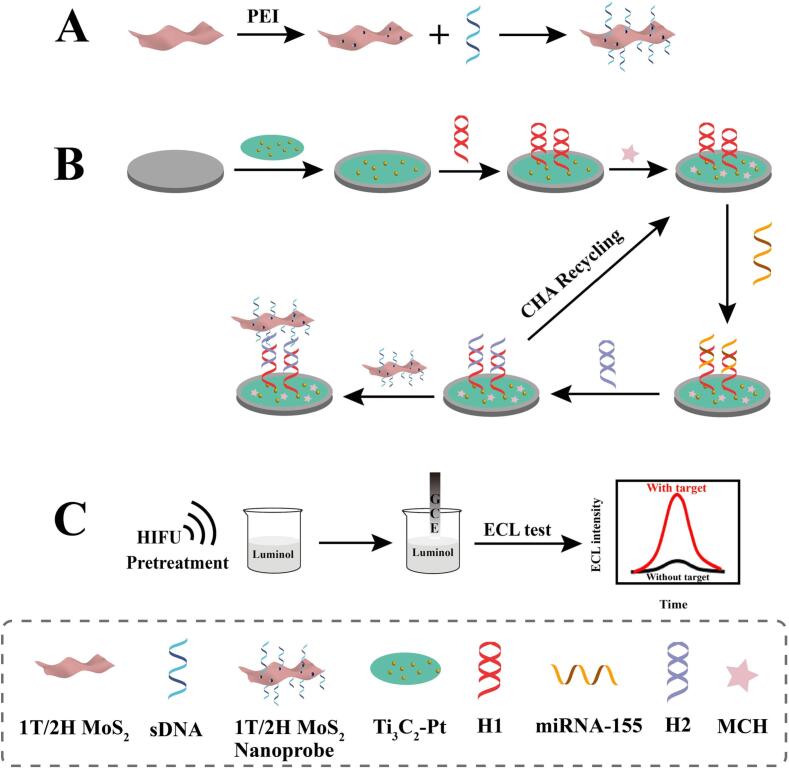
(A) Design of 1T/2H MoS_2_ nanoprobe. (B) Construction of the biosensor. (C) HIFU pretreatment and then the ECL detection.

In view of the above, we constructed a novel ECL biosensor combined the HIFU pretreatment and 1T/2H MoS_2_ for sensitive detection of miRNA-155. HIFU pretreatment promotes the conversion of dissolved O_2_ to ROS through the well-known cavitation effect, which could accelerate the oxidation of luminol and improve the ECL response. 1T/2H MoS_2_ with excellent catalytic performance accelerates the decomposition of H_2_O_2_ generated in situ, thus further enhancing the ECL emission of luminol. Scheme 1 is the design of the ECL sensor. 1T/2H MoS_2_ was modified by sDNA as a probe (Scheme 1A). Ti_3_C_2_-Pt nanomaterial was modified on glassy carbon electrode (GCE) surface to bind H1 via the Pt-S bonds. The catalytic hairpin assembly (CHA) reaction was carried out to realize the miRNA-155 cycle. Then, the modified electrode was incubated in 1T/2H MoS_2_ nanoprobe solution (Scheme 1B). Finally, ECL detection was carried out with the assistance of HIFU pretreatment (Scheme 1C). The designed ECL biosensor shows high sensitivity for miRNA-155 detection, which will provide a new prospect for the research and analysis of miRNA-155.

## 2 Experimental section

The subsection of materials, instruments, the preparation of Ti_3_C_2_-Pt, 1T/2H MoS_2_ and 1T/2H MoS_2_ nanoprobe are shown in Supplementary Material.

### 2.1 Construction of biosensor

The GCE was polished with 0.3 and 0.05 μm alumina obtaining a mirror-like surface. Electrode was ultrasonically treated in ethanol and deionized (DI) water, which was dried with nitrogen. Then, 6 μL Ti_3_C_2_-Pt was dropped onto the GCE surface and dried. Subsequently, Ti_3_C_2_-Pt/GCE was incubated in 40 μL H1 solution (2 μM) for 12 h to obtain H1/Ti_3_C_2_-Pt/GCE. After that, H1/Ti_3_C_2_-Pt/GCE was incubated in 40 μL MCH solution (0.5 mM) for 2 h to eliminate the non-specific binding effect and obtain MCH/H1/Ti_3_C_2_-Pt/GCE. Next, 20 μL H2 (2 μM) and 20 μL miRNA-155 were mixed, and CHA reaction was performed in the above mixed solution to obtain H2 + H1/MCH/Ti_3_C_2_-Pt/GCE. Finally, H2 + H1/MCH/Ti_3_C_2_-Pt/GCE was incubated in 1T/2H MoS_2_ nanoprobe solution for 2 h to obtain the 1T/2H MoS_2_/H2 + H1/MCH/Ti_3_C_2_-Pt/GCE.

### 2.2 HIFU pretreatment

HIFU pretreatment was performed in luminol solution (50 μM, pH 10.91) for 5 min with 7.5 W, and then removed before the ECL detection. Next, the modified electrode was immediately inserted into the above solution to obtain ECL emission.

## 3 Results and discussion

### 3.1 Characterization of Ti_3_C_2_ MXene and Ti_3_C_2_-Pt

**Fig. 1:**
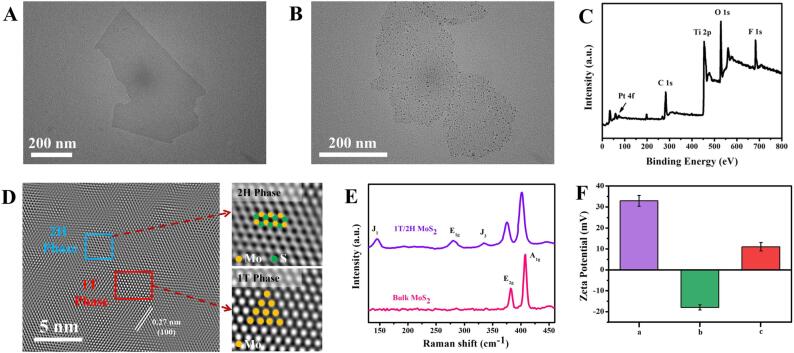
The TEM images of (A) Ti_3_C_2_ MXene and (B) Ti_3_C_2_-Pt. (C) XPS spectrum of the Ti_3_C_2_-Pt. (D) HRTEM image of 1T/2H MoS_2_. (E) The Raman spectra of bulk MoS_2_ and 1T/2H MoS_2_. (F) Zeta potential values of PEI (a), 1T/2H MoS_2_ (b) and 1T/2H MoS_2_-PEI (c).

To verify the successful synthesis of Ti_3_C_2_ MXene and Ti_3_C_2_-Pt, we performed transmission electron microscopy (TEM), dynamic light scattering (DLS), X-ray diffraction (XRD) and X-ray photoelectron spectroscopy (XPS) characterization. As shown in Fig. 1A, Ti_3_C_2_ MXene exhibits monodisperse sheet structure. The data measured by DLS (Fig. S1) proves that the size of Ti_3_C_2_ MXene is about 200 nm. Fig. S2 shows the XRD patterns of Ti_3_AlC_2_ (a), Ti_3_C_2_ (b) and Ti_3_C_2_-Pt (c). Comparing with the XRD pattern of Ti_3_AlC_2_, the (104) main peak of Ti_3_C_2_ disappears and the (002) peak moves to the left, indicating that Al is etched in Ti_3_AlC_2_. In addition, the diffraction peaks 2^θ^ ≈ 39.9°, 46.4° and 67.7° correspond to the (111), (200) and (220) planes of Pt, respectively, thus proving the successful synthesis of Ti_3_C_2_-Pt. TEM characterization (Fig. 1B) also clearly shows that Pt NPs are deposited on Ti_3_C_2_ MXene nanosheets. Ti_3_C_2_ MXene and Ti_3_C_2_-Pt are further characterized by XPS. Elements Pt, C, Ti, O and F are observed in the XPS map of Ti_3_C_2_-Pt in Fig. 1C. C, Ti, O, and F are derived from Ti_3_C_2_, and Pt is derived from Pt NPs generated in situ. The XPS pattern of Ti element in Ti_3_C_2_ MXene (Fig. S3A) shows that the peaks located at 455.3, 456.3 and 461.6 eV correspond to Ti (II), Ti-C and Ti-O bonds, respectively. As shown in Fig. S3B, when Pt NPs are introduced, Ti element is transformed from Ti (II) to Ti (IV). Meanwhile, as shown in the Pt 4f orbital spectrum (Fig. S3C), the peaks located at 71.2 eV and 74.5 eV are attributed to Pt (0) bonds, while the peaks located at 70.2 eV and 73.5 eV correspond to Pt (II). The above results indicate that Ti_3_C_2_-Pt has been successfully prepared.

### 3.2 Characterization of 1T/2H MoS_2_

The successful synthesis of 1T/2H MoS_2_ was demonstrated by scanning electron microscopy (SEM), high-resolution transmission electron microscopy (HRTEM), Raman and zeta characterization. 1T/2H MoS_2_ is a distinct sheet-like structure (Fig. S4). We chose HRTEM as a tool to detect the phase of 1T/2H MoS_2_ because of its high resolution and high definition. As shown in Fig. 1D, the 1T phase region shows the Mo-S octahedral coordination, while the 2H phase region exhibits the typical Mo-S trigonalprismatic coordination. In Raman spectrum (Fig. 1E), the bulk MoS_2_ before chemical intercalation is a completely 2H phase, thus it only shows two characteristic peaks at 380 cm^−1^ (E_2g_) and 404 cm^−1^ (A_1g_). In addition to characteristic peaks of 2H phase, exfoliated MoS_2_ has 1T structure in the Raman spectrum, which appears at around 150, 280 and 330 cm^−1^, corresponding J_1_, E_1g_, and J_3_ modes respectively [37]. The HRTEM image and Raman spectrum could prove that 1T/2H MoS_2_ was successful synthesized. 1T/2H MoS_2_ shows a negative zeta potential, while 1T/2H MoS_2_-PEI becomes electropositive of 11.01 mV, indicating the successful modification of the cationic polymer PEI (Fig. 1F).

### 3.3 Effects of HIFU pretreatment and 1T/2H MoS_2_ catalysis on luminol-O_2_ ECL

**Fig. 2:**
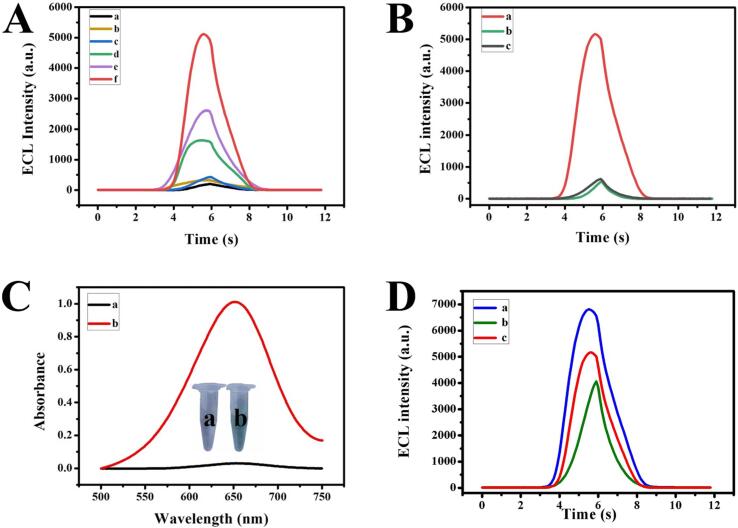
(A) ECL intensity of bare GCE (a), bulk MoS_2_/GCE (b) and 1T/2H MoS_2_/GCE (c) in luminol-O_2_ without HIFU pretreatment; ECL intensity of bare GCE (d), bulk MoS_2_/GCE (e) and 1T/2H MoS_2_/GCE (f) in luminol-O_2_ with HIFU pretreatment. (B) ECL intensity of 1T/2H MoS_2_/GCE in luminol with HIFU pretreatment variation in air-saturated (a), N_2_-saturated (b) and addition benzoquinone (BQ) (c) as radical scavengers. (C) The UV–vis analysis of the mixture solution of TMB and HRP (a), the mixture solution of TMB and HRP with HIFU pretreatment (b), and the inset is the photograph of a and b. (D) ECL intensity of 1T/2H MoS_2_/GCE in 50 μM luminol containing 30 μM H_2_O_2_ (a), 20 μM H_2_O_2_ (b) and 1T/2H MoS_2_/GCE in 50 μM luminol with HIFU pretreatment (c).

The effects of bulk MoS_2_ and 1T/2H MoS_2_ in luminol-O_2_ ECL system with HIFU pretreatment were investigated. In Fig. 2A, the lower ECL signals of bare GCE (a), bulk MoS_2_/GCE (b) and 1T/2H MoS_2_/GCE (c) were observed. After luminol solution was pretreated with HIFU, the ECL signal was significantly enhanced (curves d, e, f), and the signal value of 1T/2H MoS_2_/GCE (f) was higher than bulk MoS_2_/GCE (e). According to the above results, it is speculated that HIFU pretreatment could promote the conversion of O_2_ to ROS by cavitation effect, thus enhancing the ECL signal of luminol. Meanwhile, 1T/2H MoS_2_ catalyzes the conversion of H_2_O_2_ generated in situ to O_2_^•−^ by virtue of its high catalytic activity, which further amplifies the ECL signal of luminol. In addition, we analyzed the stability of ECL signal value of 1T/2H MoS_2_/GCE in luminol-O_2_ with HIFU pretreatment by consecutive potential scans for 10 cycles (Fig. S5). The ECL intensity almost remained unchanged, indicating its excellent stability.

With aim of proving the speculation, we verified the importance of O_2_ firstly. In Fig. 2B, ECL intensity of 1T/2H MoS_2_/GCE in luminol-O_2_ with HIFU pretreatment was the highest (curve a). We continuously injected N_2_ into luminol solution to remove O_2_, 1T/2H MoS_2_/GCE obtained extremely low ECL response (curve b), which proved that O_2_ played a vital role in luminol ECL system. In order to further confirm which kind of ROS played a key role, benzoquinone (BQ) as a scavenger was used to capture O_2_^•−^. ECL signal was significantly reduced which indicating that O_2_^•−^ produced by HIFU was crucial for the enhancement of luminol ECL (curve c). In addition, the in situ production of H_2_O_2_ by HIFU pretreatment was verified by colorimetric analysis. HRP could catalyze H_2_O_2_ to oxidation of substrate TMB into colored substance TMB^*+^, which the peak absorbance is about 650 nm. In Fig. 2C, the characteristic peak of TMB^*+^ was not shown at 650 nm in the mixed solution of TMB and HRP (curve a). Surprisingly, using the strategy of HIFU pretreatment, the color of the solution turned blue and the peak of 650 nm appeared (curve b), which suggested that the HIFU could generate H_2_O_2_ in the air saturated solution.

In the comparative experiment, H_2_O_2_ as an exogenous coreaction reagent was used in the luminol system without HIFU pretreatment (Fig. 2D). The results showed that the effect achieved by HIFU pretreatment was equivalent to the 20 ∼ 30 μM H_2_O_2_.

**Fig. 3:**
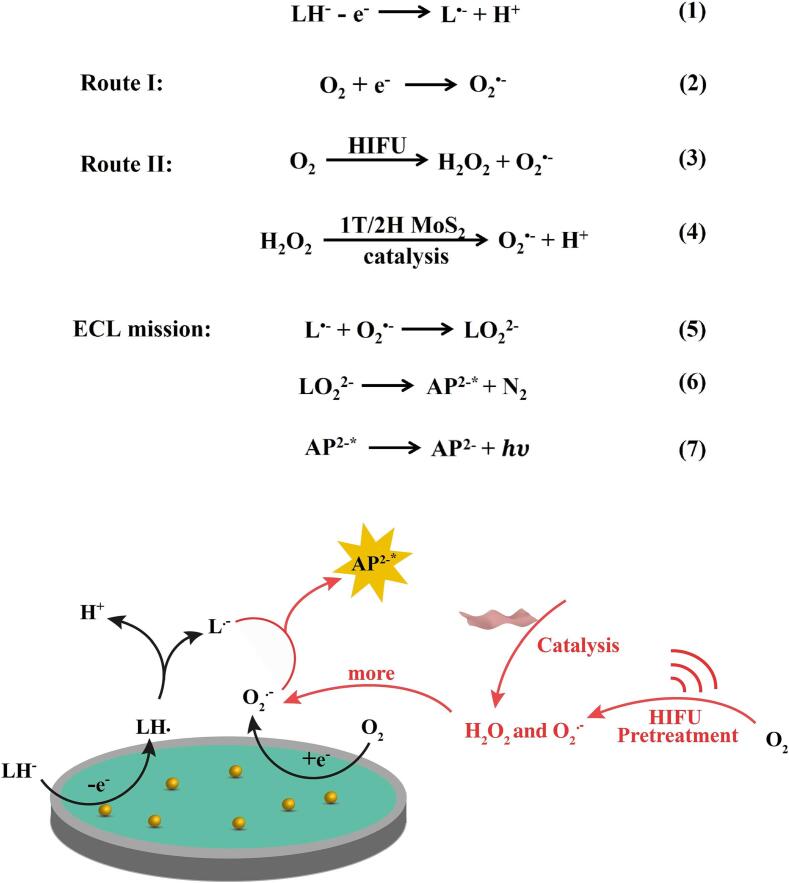
The mechanism of luminol-O_2_ ECL system with HIFU pretreatment and 1T/2H MoS_2_ catalysis.

On this basis, a possible mechanism for luminol-O_2_ ECL system based on HIFU pretreatment and 1T/2H MoS_2_ catalyze could be proposed as follows Fig. 3 and Eqs. (1) ∼ (7). First, a large amount of luminol anions (LH^−^) generate luminol anion radical (L^•−^) by electrochemical oxidation. In route I, O_2_ in the system could obtain e^−^ on the electrode to produce O_2_^•−^. In route II, HIFU pretreatment generates H_2_O_2_ and O_2_^•−^ effectively through the cavitation effect, and 1T/2H MoS_2_ catalyzes the conversion of H_2_O_2_ produced in situ to more O_2_^•−^. Finally, O_2_^•−^ interacts with the L^•−^ to produce the excited-state intermediate (AP^2−*^), which returns to the ground state to obtain significant ECL emission.

### 3.4 Electrochemical characterization of biosensor

**Fig. 4:**
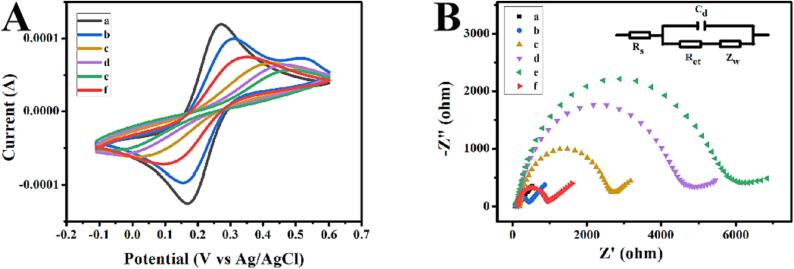
Cyclic voltammograms (A) and electrochemical impedance spectra (B) of bare GCE (a), Ti_3_C_2_-Pt/GCE (b), H1/Ti_3_C_2_-Pt/GCE (c), MCH/H1/Ti_3_C_2_-Pt/GCE (d), H2 + H1/MCH/Ti_3_C_2_-Pt/GCE (e) and 1T/2H MoS_2_/H2 + H1/MCH/Ti_3_C_2_-Pt/GCE (f) in 5 mM [Fe(CN)_6_]^3−/4−^ containing 0.1 M KCl solution. The concentration of miRNA-155 is 1 pM. The inset is the equivalent circuit of EIS.

The construction of proposed ECL sensor was characterized by cyclic voltammetry (CV) and electrochemical impedance spectroscopy (EIS). When Ti_3_C_2_-Pt was modified on the GCE, the electronic transmission of [Fe(CN)_6_]^3−/4−^ on the GCE surface was impeded, and the peak current decreased (Fig. 4A, curve b). Moreover, the peak current continuously decreased with successive decoration of H1, MCH, and H2 on the electrode surface, which could be ascribed to their non-conductivity. Finally, when the 1T/2H MoS_2_ nanoprobe was modified (curve f), the redox peak current increased owing to good conductivity of 1T/2H MoS_2_. EIS is also a good method to characterize the electrode self-assembly process (Fig. 4B). The inset is the equivalent circuit of EIS, and the four elements are internal resistance of solution (R_s_), charge-transfer resistance (R_et_), Constant phase element (C_d_) and Warburg resistances (Z_w_), respectively. With the assembly of the biosensor, the semicircle diameter gradually increased, indicating that the electron transfer resistance gradually increased. When the 1T/2H MoS_2_ nanoprobe was incubated, the resistance on the electrode surface decreased. These results correspond to those in CV, implying that this ECL sensor was constructed successfully.

### 3.5 ECL behavior of biosensor

**Fig. 5:**
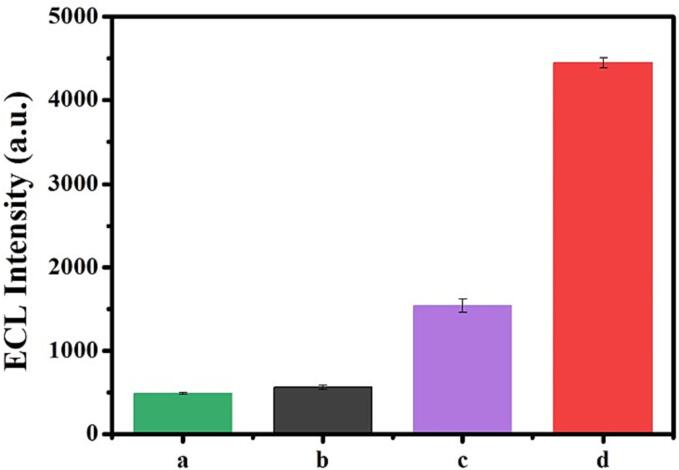
ECL intensity H2 + H1/MCH/Ti_3_C_2_-Pt/GCE (a), 1T/2H MoS_2_/H2 + H1/MCH/Ti_3_C_2_-Pt/GCE (b) in luminol-O_2_ without HIFU pretreatment; H2 + H1/MCH/Ti_3_C_2_-Pt/GCE (c) and 1T/2H MoS_2_/H2 + H1/MCH/Ti_3_C_2_-Pt/GCE (d) in luminol-O_2_ with HIFU pretreatment, the concentration of miRNA-155 is 1 pM.

The effects of HIFU pretreatment and the 1T/2H MoS_2_ were demonstrated, and shown in Fig. 5. Compared with H2 + H1/MCH/Ti_3_C_2_-Pt/GCE (a) and 1T/2H MoS_2_/H2 + H1/MCH/Ti_3_C_2_-Pt/GCE (b) without HIFU pretreatment, the higher ECL signal in luminol-O_2_ solution with HIFU pretreatment was observed at the H2 + H1/MCH/Ti_3_C_2_-Pt/GCE (c) and 1T/2H MoS_2_/H2 + H1/MCH/Ti_3_C_2_-Pt/GCE (d). This is mainly attributed to the fact that HIFU could generate ROS effectively by virtue of its excellent cavitation effect. Comparing with (c), (d) showed higher ECL emission, which thanks to its catalytic effect of 1T/2H MoS_2_ on H_2_O_2_. The synergistic effects of the HIFU pretreatment and the 1T/2H MoS_2_ result in amplification for ECL signal in luminol-O_2_ system.

### 3.6 Optimization of experimental conditions

We optimized the CHA reaction time and probe incubation time in a view to obtaining the best experimental results. As displayed in Fig. S6A, the strongest ECL signal was obtained when CHA reaction time reached 2 h, indicating that the CHA reaction had reached saturation. If we continue to increase the reaction time, ECL signal will decrease instead. The incubation time of the 1T/2H MoS_2_ probe was carried out (Fig. S6B), when the probe incubation time was 2 h, the ECL intensity reached the maximum, thus proving the hybridization reaction of H2 reached saturation at 2 h. In the experiment, the optimal time for CHA reaction is 2 h, and the optimal time for probe incubation is 2 h.

### 3.7 ECL detection of miRNA-155

**Fig. 6:**
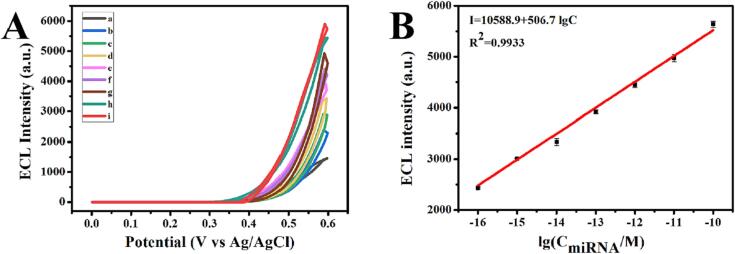
(A) ECL intensity-potential curves for various concentrations of miRNA-155 (0 fM, 0.1 fM, 1.0 fM, 10 fM, 100 fM, 1.0 pM, 10 pM, 100 pM, 1 nM (from a to i)). (B) The linear relationship between the ECL intensity and the logarithmic value of miRNA-155 concentration.

Upon the above optimization conditions, the developed detection strategy was applied for quantitative analysis of miRNA-155. As indicated from Fig. 6A, the ECL intensity was continuously amplified with the increase of target concentration. A satisfying linear relationship between the concentration of miRNA-155 from 0.1 fM to 100 pM and the ECL signal was obtained (Fig. 6B). The linear regression equation is *I_ECL_* = 10588.9 + 506.7lg*C_miRNA-155_*, and the correlation coefficient is 0.9933. Meanwhile, the limit of detection (LOD) is 0.057 fM calculated by LOD = 3σ/K (σ represents the background standard deviation obtained from 10 parallel experiments and K represents the slope of the regression equation). In addition, we summarized the analytical performance of the reported methods and listed in the Table S1. The comparison shows that our work has a higher sensitivity and wider linear range.

### 3.8 Selectivity and stability of biosensor

**Fig. 7:**
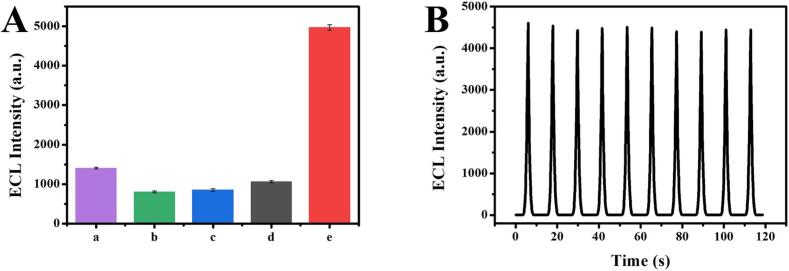
(A) Selectivity of the designed ECL biosensor: blank (a), noncomplementary DNA (b), noncomplementary RNA (c), single-base difference RNA (d) and miRNA-155 (e). (B) Stability test of the designed ECL biosensor, the concentration of miRNA-155 is 1 pM.

Selectivity and stability play an important role in evaluating ECL sensing performance. In Fig. 7A, the ECL responses of the biosensor for blank solution, non-complementary DNA (100 pM), non-complementary RNA (100 pM) and single-base difference RNA (100 pM) were weak, while it was strong to miRNA-155 (10 pM), demonstrating the wonderful selectivity of the ECL biosensor. The stability of the sensing platform was analyzed by consecutive potential scans for 10 cycles (Fig. 7B). The ECL intensity had no obvious variation and the RSD was 0.80%, thus indicating the excellent stability for the proposed ECL sensing platform.

### 3.9 Analysis of miRNA-155 in actual samples

We performed a recovery test in human serum to study the feasibility of the designed ECL biosensor. By calculation, the recovery rates were 97.72 % ∼ 104.71 % with the RSD in the range of 2.70 % ∼ 7.27 % (Table 1). Thus, the designed ECL biosensing platform could be used in human samples to determine RNA, and have a good prospect in clinical application.

**Table 1:** Determination of miRNA-155 in human serum samples.

Sample	Added (M)	Found (M)	RSD (%)	Recovery (%)
1	1.00×10^−15^	1.05×10^−15^	3.96	104.71
2	1.00×10^−14^	1.02×10^−14^	7.27	101.98
3	1.00×10^−13^	9.77×10^−14^	6.75	97.72
4	1.00×10^−12^	9.91×10^−13^	2.70	99.08

## 4 Conclusion

In summary, we constructed a highly sensitive ECL biosensing platform, which combined the HIFU pretreatment, 1T/2H MoS_2_ and CHA reaction to detect and analyze miRNA-155. In this strategy, HIFU pretreatment generates ROS in situ to improve the ECL signal of luminol, meanwhile 1T/2H MoS_2_ has good conductivity and electrocatalytic activity further catalyze the H_2_O_2_ generated in situ to enhance the ECL response. The ECL biosensing platform has excellent selectivity, stability, reproducibility, and sensitivity. This study combining ultrasound with physical materials proposes a new way to improve the ECL response of luminol-O_2_ system, and also brings a new strategy for miRNA-155 ultra-sensitive detection, which will inspire the development of novel ECL platforms in the future.

## Declaration of Competing Interest

The authors declare that they have no known competing financial interests or personal relationships that could have appeared to influence the work reported in this paper.

## Data Availability

No data was used for the research described in the article.
