# Supplementary material for: Boosting the electrochemiluminescence of luminol by high-intensity focused ultrasound pretreatment combined with 1T/2H MoS2 catalysis to construct a sensitive sensing platform
Source: Ultrason Sonochem. 2022 Dec 12;92:106264. doi: 10.1016/j.ultsonch.2022.106264 (PMC9768369; doi:10.1016/j.ultsonch.2022.106264)
Supplement: Supplementary data 1 [file mmc1.docx]

**Supplementary Material**

**Boosting the electrochemiluminescence of luminol by high-intensity focused ultrasound pretreatment combined with 1T/2H MoS_2_ catalysis to construct a sensitive sensing platform**

Lin Du, Huixin Zhang, Zhenyu Wang, Tingting Zhuang, Zonghua Wang*

*College of Chemistry and Chemical Engineering, Shandong Sino-Japanese Centre for Collaborative Research of Carbon Nanomaterials, Instrumental Analysis Centre of Qingdao University, Institute of Biomedical Engineering, Qingdao University, Qingdao, Shandong, 266071, China.*

*Corresponding author

E-mail: [wangzonghua@qdu.edu.cn](mailto:wangzonghua@qdu.edu.cn)

**Table of Contents**

**Materials ----------------------------------------------------------------------- S3**

**Instruments ------------------------------------------------------------------- S3**

**Preparation of Ti_3_C_2_-Pt ----------------------------------------------------- S4**

**Preparation of 1T/2H MoS_2_ -****---------------------------------------------- S5**

**Preparation of 1T/2H MoS_2_ nanoprobe --------------------------------- S5**

**Fig. S1. The DLS characterizations of Ti_3_C_2_ MXene ----------------- S6**

**Fig. S2. The XRD characterizations ------------------------------------- S6**

**Fig. S3. The XPS characterizations -------------------------------------- S6**

**Fig. S4. The SEM characterizations of 1T/2H MoS_2_ ----------------- S7**

**Fig. S5. The stability of 1T/2H MoS_2_/GCE in luminol-O_2_ with HIFU pretreatment ------------------------------------------------------------------ S7**

**Fig. S6. The optimization of the ECL biosensor ----------------------- S7**

**Table S1. Comparison of the developed with reported methods for determination of miRNA-155 --------------------------------------------- S8**

**Materials**

Ti_3_AlC_2_ (98%) was purchased from Forsman Scientific Co., Ltd. (Beijing, China). MoS_2_ powder, n-hexane, n-butyllithium (n-Buli), mercaptoethanol (MCH), hydrogen peroxide (30%), and luminol were purchased from Sigma-Aldrich. NaBH_4_ was obtained from Shanghai Sinopharm. Benzoquinone (BQ) and Polyethylene imine (PEI, MW=70000) was purchased from Shanghai maclin biochemical co., LTD. 1-(3-(dimethylamino) propyl)-3-ethyl carbondiimide hydrochloride (EDC), N-hydroxysuccinimide (NHS), lithium fluoride (LiF) and H_2_PtCl_6_·6H_2_O were obtained from Shanghai Aladdin Biochemical Technology Co., LTD. The oligonucleotides used were synthesized and purified by Shanghai Sangon Biological Engineering Technology & Services Co., Ltd. The sequences of oligonucleotides were listed in Table S1.

**Table S1.** Sequences of the used oligonucleotides in this work.

| Name | Sequences (5’ to 3’) |
| --- | --- |
| sDNA | COOH - TTA GTC GCT CCT |
| Target (miRNA-155) | UUA AUG CUA AUC GUG AUA GGG GU |
| H1 | TAA TCG TGA TAG GGG TAT GGA CAT GGA ACC CCT ATC ACG ATT AGC ATT AAA GA- SH |
| H2 | ATG GAC ATG GAT AAT CGT GAT AGG GGT CCC ATG TCC ATA CCC CTA TGA AGG AGC GAC T |
| Noncomplementary DNA (nDNA) | GGT TGG TGT GGT TGG |
| miRNA-101 (nRNA) | UAC AGU ACU GUG AUA ACU GAA |
| Single-base difference miRNA (sRNA) | UUA AGG CUA AUC GUG AUA GGG GU |

**Instruments**

Scanning electron microscopy (SEM) measurements were performed using a Hitachi S-4800 field-emission scanning electron microscope. High-resolution transmission electron microscopy (HRTEM, FEI Tecnai G2 F20, USA). X-ray diffraction (XRD) patterns were recorded on a Bruker D8 ADVANCE diffractometer. The X-ray photoelectron spectroscopy (XPS) measurements were investigated using monochromatized AlK α (1486.6 eV) X-ray source in Thermo ESCALAB 250XI apparatus (Thermo Fisher, USA). HRTEM was performed on a Thermo Fisher Scientific Titan Themis Z to observe the device architecture and crystal structure. The acceleration voltage was 300 kV. The Raman spectrum was obtained by DXR2 Microscopic Raman spectrometer (Thermo Fisher, USA). The charge detection and dynamic light scattering analysis of the material were carried out using a 90 Plus Zeta potential and particle size analyzer (Brookhaven Instruments, USA). The UV-Vis characterization was carried out using the UV-2700 (Shimadzu Co., Kyoto, Japan). Cyclic voltammetry (CV) and electrochemical impedance spectroscopy (EIS) experiments were performed on a Princeton Electrochemical Workstation (Parstat Mc). Electrogenerated chemiluminescence was performed with MPI-E electrochemiluminescence analyzer detector (Xi’an Remex analysis Instrument Co, Ltd., China). The voltage of the photomultiplier tube (PMT) was maintained at 600 V. The CV test was performed in a 5 mM K_3_Fe(CN)_6_/K_4_Fe(CN)_6_ solution containing 0.1 M KCl with a canning rate of 0.1 V/s and a potential range of -0.2~0.6 V. EIS was performed in a 5 mM K_3_Fe(CN)_6_/K_4_Fe(CN)_6_ solution containing 0.1 M KCl in the frequency range of 10^-2^~10^5^ Hz under a signal amplitude of 5 mV. HIFU pretreatment was performed on Sonic-Stimu Basic (UT1021, NU-TK) with 1.0 MHz, and the duty cycle was 70 %.

**Preparation of Ti_3_C_2_-Pt**

0.8 g LiF was added to 10 mL HCl (9 M) and stirred for 5 min, then add 0.5 g Ti_3_AlC_2_ powder to the mixed solution and stir at 35°C for 24 h. After that, the suspension was centrifuged several times and the solids were separated from the supernatant. Then, the solid precipitate was added to deionized water (DI), and N_2_ was injected into the solution, and the solution was sonicated for 1 h. Finally, the solution was centrifuged at 3500 rmp, and the supernatant was collected and passed into nitrogen to reserve Ti_3_C_2_ MXene. 5 mL Ti_3_C_2_ (0.234 mg/mL) solution was ultrasonically treated for 5 min, followed by the addition of 250 μL H_2_PtCl_6_ (10 mg/mL) with continuous ultrasonication. 10 μL NaBH_4_ (10 mM) was added to the mixture and stirred vigorously for 2 h. Finally, the solution was centrifuged, the precipitate was retained and redispersed in distilled water to obtain Ti_3_C_2_-Pt solution.

**Preparation of 1T/2H MoS_2_**

0.5 g MoS_2_ was placed in the Teflon liner and vacuum dried overnight at 100°C in the oven. 5 mL n-hexane and 4 mL n-BuLi (2 M) were added to a high pressure reaction kettle containing MoS_2_, and reacted at 65°C for 48 h in an atmosphere of argon. Approximately 100 mL DI water was added to the cooled reaction mixture under hydrogen evolution. After the amount of gas production was reduced, the organic impurities were removed by extraction with n-hexane and the aqueous phase was collected. The resulting solution was sonicated in a water bath for 6 hours. After that, unstripped MoS_2_ was removed by centrifugation at 3000 rmp, very small MoS_2_ material and LiOH were removed by centrifugation at 8000 rmp and 12000 rmp, and the pH in the supernatant gradually changed from 14 to 7. Finally, the sediment was redispersed in DI water by bath sonication, yielding a highly stable dispersion.

**Preparation of 1T/2H MoS_2_ nanoprobe**

The mixed solution of 2 mL 1T/2H MoS_2_ (0.023 mg/mL) and 1 mL PEI (2.5 mg/mL) was sonicated for 10 min and stirred for 2 h. Subsequently, the mixture was centrifuged and washed several times to obtain 1T/2H MoS_2_-PEI by retaining the precipitate. Then, EDC (400 mM) and NHS (100 mM) were reacted with 60 μL sDNA solution (1 μM) at 37°C for 1.5 h, and 200 μL of 1T/2H MoS_2_-PEI solution was added and stirred for 1 h. After that, the mixed solution was centrifuged at 12000 rmp. Finally, the sediment was redispersed in deionized water (DI) to obtain 1T/2H MoS_2_ nanoprobe for the following experiments.


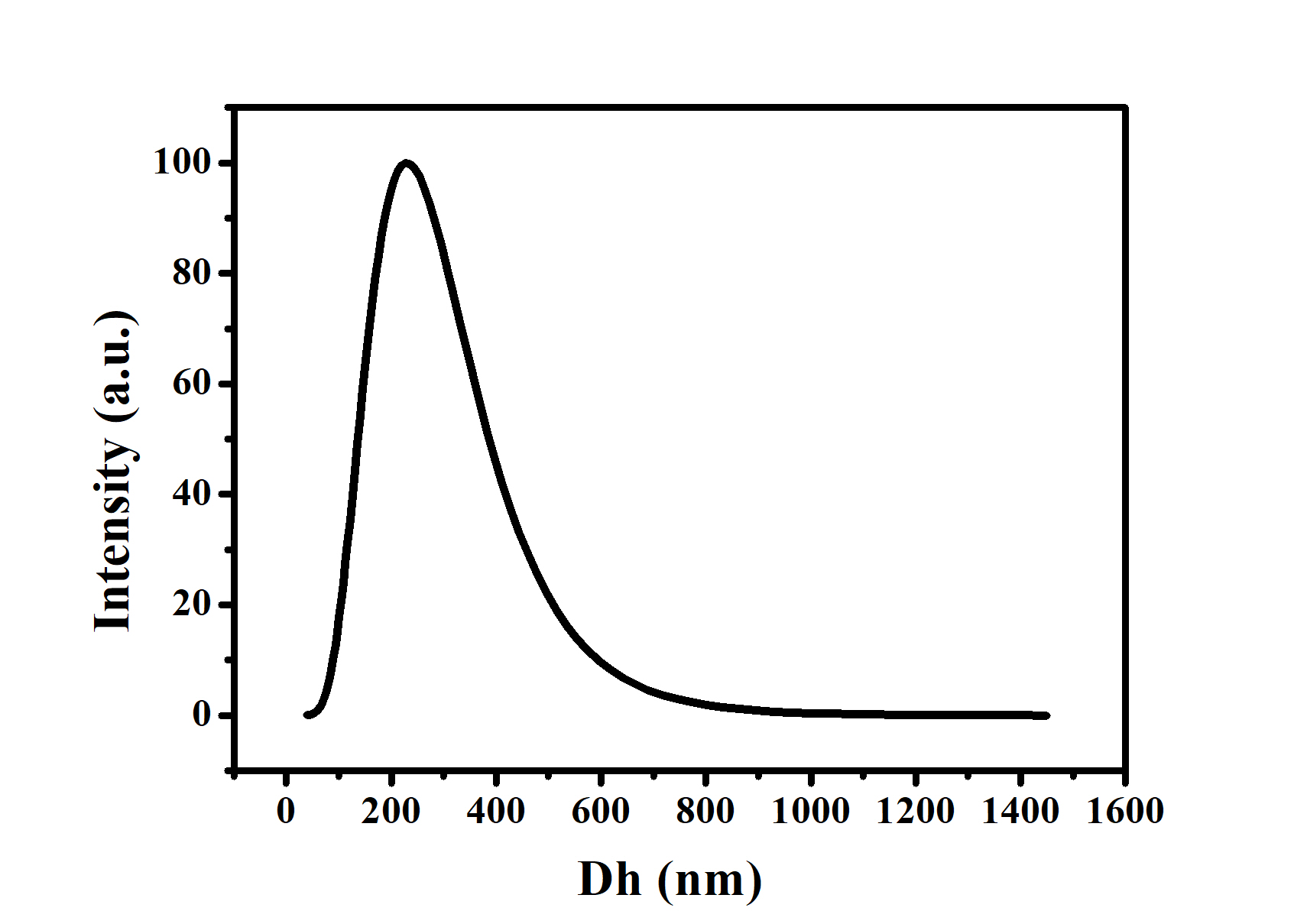


**Fig. S1** The size distribution histogram of Ti_3_C_2_ MXene.


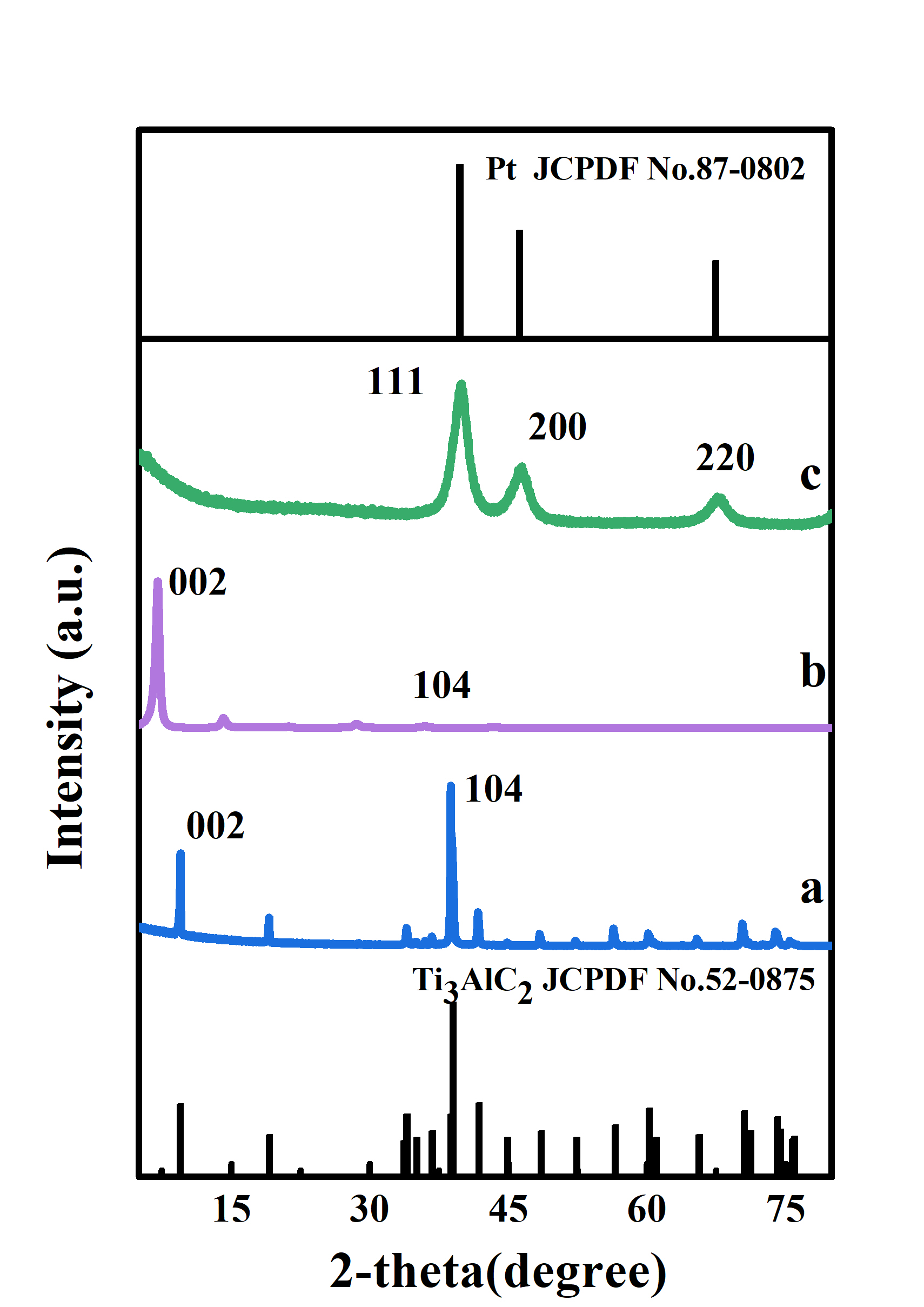


**Fig. S2** The XRD patterns of the Ti_3_AlC_2_ (a), Ti_3_C_2_ MXene (b), Ti_3_C_2_-Pt (c).


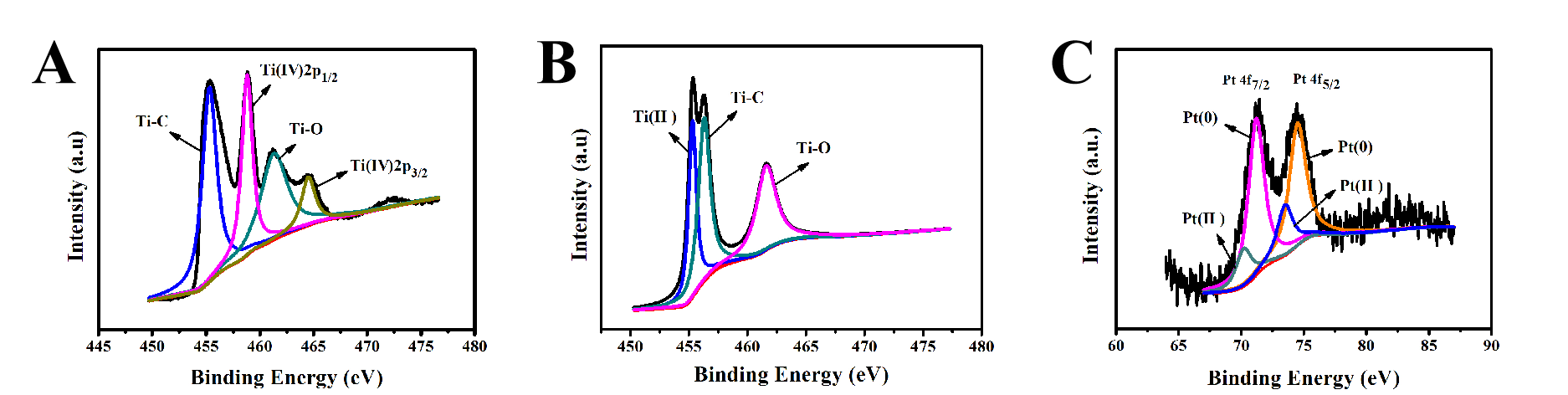


**Fig. S3** (A) The XPS spectrum of Ti element in the Ti_3_C_2_ MXene. (B) The XPS spectrum of Ti element and (C) the XPS spectrum of Pt element in the Ti_3_C_2_-Pt.


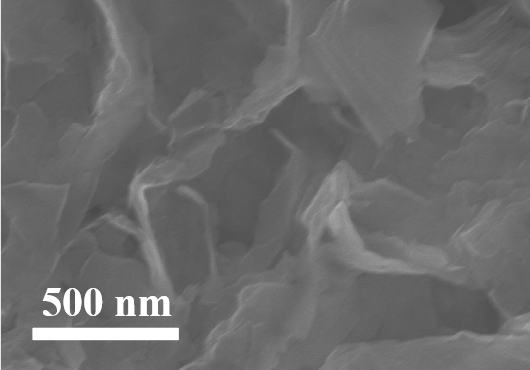


**Fig. S4** The SEM of 1T/2H MoS_2_.


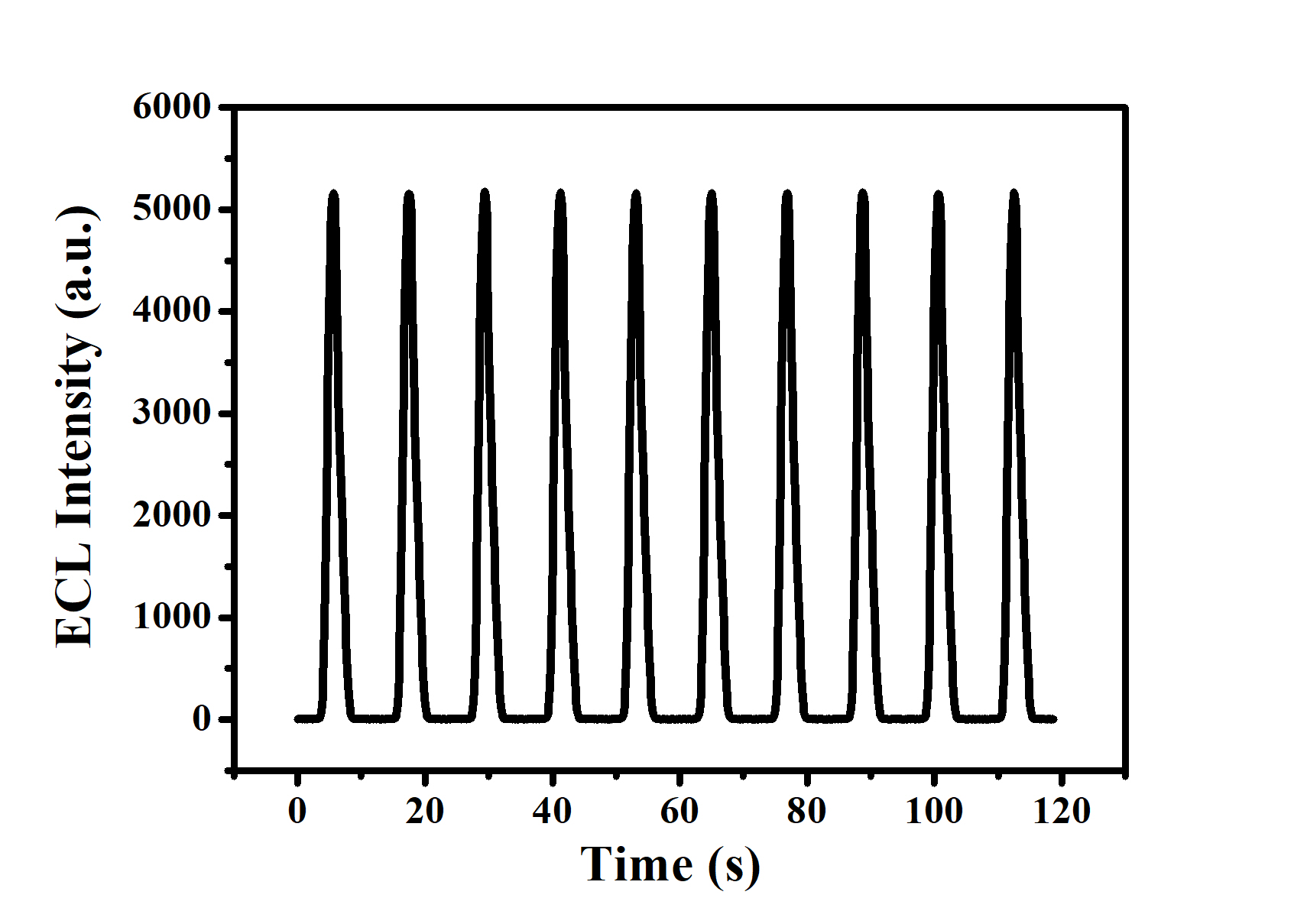


**Fig. S5** The stability of 1T/2H MoS_2_/GCE in luminol-O_2_ with HIFU pretreatment.


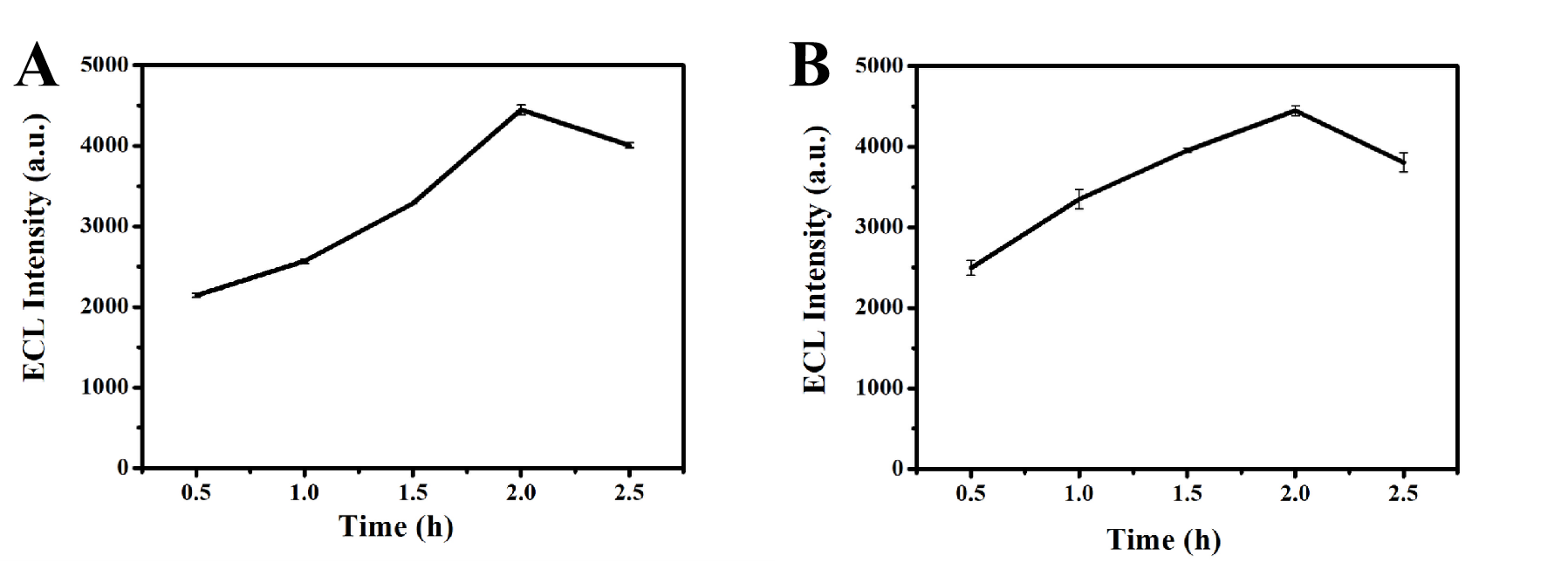


**Fig. S6** ECL intensity of the biosensor tested with different time of CHA reaction (A) and different time of 1T/2H MoS_2_ probe incubation (B). The concentration of miRNA-155 is 1 pM. The error bars were calculated from three times parallel experiments.

**Table S1**

Comparison of the developed with reported methods for determination of miRNA-155.

| Method | Linear range | LOD | Ref. |
| --- | --- | --- | --- |
| Electrochemistry | 0.5 fM ~ 1 nM | 0.39 fM | [1] |
| Colorimetric | 1 nM ~100 nM | 0.7 nM | [2] |
| Fluorescence | 10 fM ~1 nM | 6.28 fM | [3] |
| ECL | 0.8 fM ~1.0 nM | 0.3 fM | [4] |
| ECL | 1.0 fM ~ 500 pM | 0.45 fM | [5] |
| ECL | 0.1 fM ~ 1 nM | 0.057 fM | This work |

**References**

[1] Y. Zhao, C.T. Lu, X.E. Zhao, W.H. Kong, S.Y. Zhu, F.L. Qu, A T-rich nucleic acid-enhanced electrochemical platform based on electroactive silver nanoclusters for miRNA detection, Biosensors and Bioelectronics 208 (2022).

[2] K. Shahsavar, E. Shokri, M. Hosseini, Sensitive colorimetric detection of miRNA-155 via G-quadruplex DNAzyme decorated spherical nucleic acid, Microchimica Acta 189 (2022).

[3] J.J. Wang, Y. Liu, Z. Ding, L. Zhang, C.Q. Han, C.C. Yan, E. Amador, L.Q. Yuan, Y. Wu, C.Y. Song, The exploration of quantum dot-molecular beacon based MoS_2_ fluorescence probing for myeloma-related Mirnas detection, Bioactive Materials 17 (2022) 360-368.

[4] Y.N. Jian, H. Wang, F.F. Lan, L.L. Liang, N. Ren, H.Y. Liu, S.G. Ge, J.H. Yu, Electrochemiluminescence based detection of microRNA by applying an amplification strategy and Hg(II)-triggered disassembly of a metal organic frameworks functionalized with ruthenium(II)tris(bipyridine), Microchimica Acta 185 (2018).

[5] B. Shen, Q. Wu, Y.P. Fan, H.P. Wu, X.M. Li, X.F. Zhao, Y.W. Wang, S.J. Ding, J. Zhang, TiO_2_@Ag nanozyme enhanced electrochemiluminescent biosensor coupled with DNA nanoframework-carried emitters and enzyme-assisted target recycling amplification for ultrasensitive detection of microRNA, Chemical Engineering Journal 445 (2022).
